# Magnesium Enhances Immune Clearance of *P. gingivalis* in Macrophages by Suppressing MCU‐Mediated Mitochondrial Calcium Overload

**DOI:** 10.1111/cpr.70282

**Published:** 2026-09-25

**Authors:** Dao‐Kun Deng, Meng‐Jie Su, Min‐Yi Zhang, Yu‐Jie Tan, Wenyao Kongling, Hong‐Yu Wang, Mei Xu, Shi‐Yu Liu, Xiao‐Tao He, Fa‐Ming Chen, Bei‐Min Tian, Xuan Li

**Affiliations:** ^1^ State Key Laboratory of Oral & Maxillofacial Reconstruction and Regeneration, National Clinical Research Center for Oral Diseases, Shaanxi International Joint Research Center for Oral Diseases, Department of Periodontology, School of Stomatology The Fourth Military Medical University Xi'an Shaanxi China; ^2^ Military Medical Innovation Center Fourth Military Medical University Xi'an Shaanxi China; ^3^ Department of Periodontal and Oral Mucosa Changsha Stomatological Hospital Changsha China; ^4^ Key Laboratory of Shaanxi Province for Craniofacial Precision Medicine Research College of Stomatology, Xi'an Jiaotong University Xi'an China; ^5^ State Key Laboratory of Oral & Maxillofacial Reconstruction and Regeneration, National Clinical Research Center for Oral Diseases, Shaanxi Key Laboratory of Stomatology, Department of Oral Biology School of Stomatology, The Fourth Military Medical University Xi'an China

**Keywords:** immune escape, macrophage, magnesium, mitochondrial calcium uniporter, *P. gingivalis*, periodontitis

## Abstract

*P. gingivalis* , a keystone periodontal pathogen in periodontitis, is recognized for its capacity to evade host immune clearance. Its intracellular survival leads to both local and systemic infection, thereby increasing the risk of periodontitis and its associated systemic diseases. However, macrophages have limited capacity to clear *P. gingivalis* , which contributes to disease establishment and progression. Our findings confirmed *P. gingivalis* presence in periodontitis tissue, with intracellular *P. gingivalis* mainly located in macrophages. However, *P. gingivalis* cannot be completely cleared by macrophages and enters the blood circulation through damaged periodontal tissue, leading to systemic infection. Transcriptomic analysis identified mitochondrial calcium uniporter (MCU) as a potential key molecule associated with *P. gingivalis* immune evasion in macrophages. In vitro functional analyses further confirmed that *P. gingivalis* infection increased MCU expression and led to both cytosolic and mitochondrial calcium overload, thereby impairing intracellular *P. gingivalis* clearance in macrophages. Notably, as a natural calcium antagonist, magnesium could inhibit the MCU pathway, reverse calcium overload, and enhance the macrophage‐mediated *P. gingivalis* clearance. Local administration of magnesium‐containing alginate methacryloyl (AlgMA) hydrogel significantly reduced both local and systemic *P. gingivalis* infection, relieved inflammation, and alleviated periodontal tissue destruction. Collectively, our findings identify MCU‐mediated calcium overload as a previously unrecognized mechanism by which *P. gingivalis* evades macrophage immune clearance. Moreover, magnesium‐mediated restoration of Ca^2+^ homeostasis offers a promising host‐directed immunomodulatory therapy for periodontitis and other infectious diseases.

## Graphical abstract

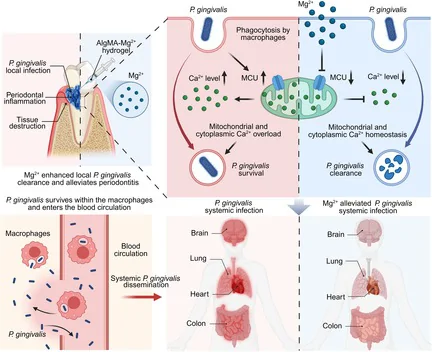

## 1 Introduction

Periodontitis is a common chronic inflammatory condition marked by the gradual breakdown of tissues supporting the teeth, which can eventually result in tooth loss in adults [1]. Mounting evidence suggests that periodontitis is more than just a localized oral condition; it poses a potential risk for systemic diseases, including cardiovascular, respiratory, Alzheimer's, intestinal disorders, and cancer [2, 3, 4]. In periodontitis and its related systemic illnesses, *P. gingivalis* is identified as a primary pathogen due to its capacity to evade host immune clearance and drive both local and systemic infection [5, 6].

As key elements of the innate immune system, macrophages and neutrophils are essential for the phagocytosis and clearance of *P. gingivalis* . However, *P. gingivalis* employs various strategies to avoid host immune clearance and persist within innate immune cells [7, 8]. For instance, previous studies demonstrated that *P. gingivalis* not only exploits the complement system [9, 10], but also regulates the TLR2 signalling pathway, thereby impairing the phagocytosis and clearance of *P. gingivalis* in macrophages and neutrophils [9, 11]. These mechanisms cause both local periodontal infection and systemic infection, driving the progression of periodontitis and its related systemic illnesses [12, 13, 14]. Therefore, elucidating the *P. gingivalis* immune evasion mechanism is of great significance for developing novel therapies for periodontitis and other *P. gingivalis* ‐related diseases.

Despite progress in elucidating *P. gingivalis* immune evasion mechanism [15, 16, 17], the mechanisms by which *P. gingivalis* evades intracellular clearance remain incompletely understood. Calcium ions function as a second messenger within cells, crucial for *P. gingivalis* phagocytosis and clearance [18]. Calcium signalling is involved in the regulation of phagocytosis, phagosome maturation, and intracellular bacterial killing [19, 20, 21, 22, 23]. Notably, the mitochondrial calcium uniporter (MCU) is the major channel responsible for Ca^2+^ uptake into the mitochondrial matrix, playing a central role in cellular Ca^2+^ homeostasis [24, 25]. MCU dysfunction can impair mitochondrial function and aggravate inflammatory responses in macrophages during periodontitis [26, 27]. In periodontitis, *P. gingivalis* evades immune clearance and persists within host cells, thereby leading to periodontal tissue destruction and systemic dissemination [28]. However, it remains unknown whether *P. gingivalis* manipulates the MCU to regulate calcium levels, thereby influencing its survival, local infection, and systemic dissemination.

Magnesium ions not only act as a natural calcium antagonist to regulate intracellular calcium levels, but also modulate the function of immune cells, emerging as a promising candidate for host‐directed immunomodulatory therapy in bacterial infectious disease [29, 30]. In this study, we first demonstrated that *P. gingivalis* was mainly phagocytosed by macrophages but could not be completely cleared in a rat model of periodontitis. To investigate the mechanism by which *P. gingivalis* escaped macrophage‐mediated immune clearance, we performed transcriptomic analysis and identified the mitochondrial calcium uniporter (MCU) as a critical player involved in *P. gingivalis* immune evasion. Subsequent in vitro functional experiments confirmed that MCU and calcium overload are key mechanisms underlying the *P. gingivalis* immune evasion in macrophages. Finally, we evaluated whether magnesium could reverse calcium overload to enhance the macrophage‐mediated *P. gingivalis* clearance, and assessed its effects on local and systemic *P. gingivalis* infection, as well as on periodontal inflammation and tissue destruction. Together, our study identifies MCU‐mediated calcium overload as a previously unrecognized mechanism for the immune evasion of *P. gingivalis* , and provides a basis for magnesium‐based host‐directed immunomodulatory therapy as a potential strategy for treating periodontitis and other infectious diseases.

## 2 Results

### 2.1 Periodontitis Is Characterized by Local Infection and Systemic Spread of *P. gingivalis*

**FIGURE 1:**
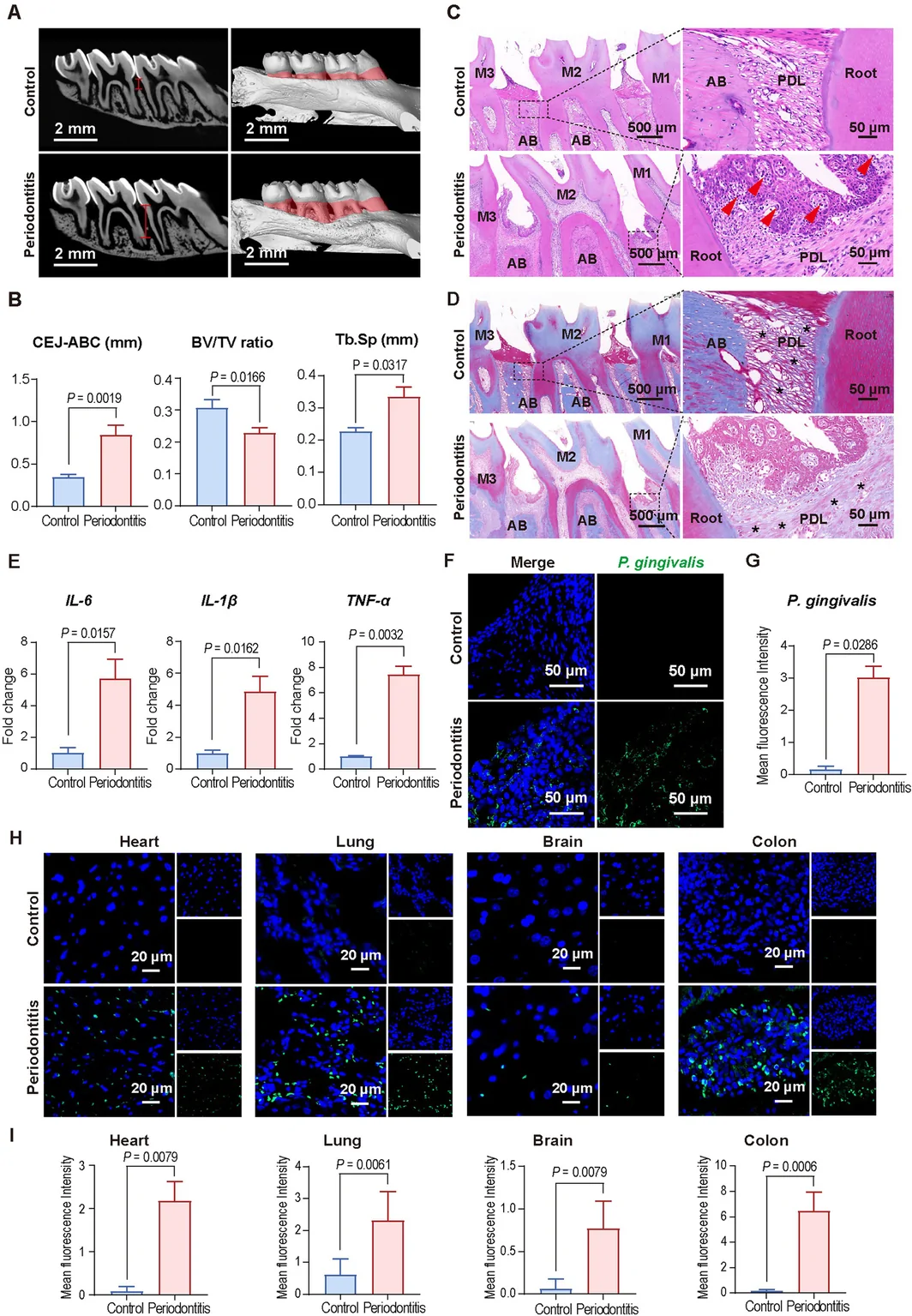
Constructing rat periodontitis model and determining the local and systemic *P. gingivalis* infection (A) representative sectioned micro‐CT images (left) and three‐dimensional reconstructions (right) of the maxillary bone in the control and periodontitis groups. Red lines represent the distance from the cementoenamel junction (CEJ) to the alveolar bone crest (ABC) of the second molars (M2) at the mesial sites. The red curve represents the ABC and the CEJ. (B) Quantification of the CEJ‐ABC distance, bone volume/total volume (BV/TV), and trabecular separation (Tb. Sp) of the maxillary M2 in control and periodontitis groups. (C) Representative H&E staining of periodontal tissue in control and periodontitis groups. Red arrowheads indicate the inflammatory cells. Periodontal ligament (PDL). Alveolar bone (AB). First molar (M1). Third molar (M3). (D) Representative Masson staining of periodontal tissue in control and periodontitis groups. Black asterisks indicate the PDL. (E) The *IL‐6*, *IL‐1β*, and *TNF‐α* gene expressions of gingiva tissues dissected from the M2 at the mesial sites in control and periodontitis groups. (F) Representative immunofluorescence (IF) images depicting the *P. gingivalis* ‐related signals within gingival tissues in control and periodontitis groups. (G) Quantification of the *P. gingivalis* mean fluorescence intensity (MFI). (H) Representative IF images showing the systemic dissemination of *P. gingivalis* ‐related signals in heart, lung, brain and colon tissues between control and periodontitis groups. (I) Quantification of the MFI. Data are represented as mean ± SD. Student's t‐test was employed with equal variances, Welch's t‐test was used for unequal variances, and Mann–Whitney U test was employed for non‐normal distribution (B, E, G, I).

To assess whether local and systemic *P. gingivalis* infection were present in periodontitis, we established a rat periodontitis model by ligating the second molars (M2) and administering *P. gingivalis* injections for 8 weeks. Rats subjected to ligation and injection served as the periodontitis group, while untreated rats served as the control group. Micro‐CT analysis depicted that ligature increased the distance from the cementoenamel junction (CEJ) to the alveolar bone crest (ABC) in the periodontitis group (Figure 1A,B). The periodontitis group also showed a reduction in bone volume to total volume ratio (BV/TV), along with increased trabecular separation (Tb.Sp) (Figure 1B). H&E staining depicted that the fibres in the periodontal ligament (PDL) were dense, intact, and organized in the control group, while the fibres in PDL were broken with inflammatory cells infiltration in the periodontitis group (Figure 1C). Masson staining showed that the PDL fibres in the periodontitis group were irregularly arranged and immature (blue). In contrast, the PDL fibres in the control group were regularly arranged and mature (red) (Figure 1D). qRT‐PCR analysis of gingival tissues dissected from the M2 revealed the increased pro‐inflammatory *IL‐6*, *IL‐1β*, and *TNF‐α* gene expression in the periodontitis group (Figure 1E). These findings showed that the ligature led to periodontal tissues destruction and inflammatory responses, indicating that the periodontitis model was successfully established.

*P. gingivalis* is commonly found in the subgingival plaque and recognized as the main factor leading to periodontitis. *P. gingivalis* was able to invade periodontal tissues, escape from immune system clearance, and further aggravate periodontitis [31]. Thus, we investigated the local presence of *P. gingivalis* or its related components in periodontal tissues using immunofluorescence (IF) and observed an increase in *P. gingivalis* ‐related fluorescent signals within gingival tissues from the periodontitis group (Figure 1F,G), indicating that *P. gingivalis* cannot be completely cleared in periodontitis tissue and leads to persistent inflammation and periodontal tissue destruction.

Beyond its detrimental effects on periodontal health, *P. gingivalis* and its products can enter the blood circulation via periodontal tissues and contribute to the development of systemic illnesses like cardiovascular, respiratory, Alzheimer's, intestinal disorders [12, 13, 14]. Thus, we assessed the systemic dissemination of *P. gingivalis* or its related components in the heart, lung, brain, and colon tissues. *P. gingivalis* ‐related signals were increased in all examined organs of periodontitis group (Figure 1H,I). These findings indicate that *P. gingivalis* or its related components cannot be cleared in periodontitis tissue and enter the blood circulation through damaged periodontal tissue, leading to systemic infection.

### 2.2 *P. gingivalis* Is Phagocytosed by Macrophages and Triggers Inflammatory Activation Accompanied by Upregulation of the MCU

**FIGURE 2:**
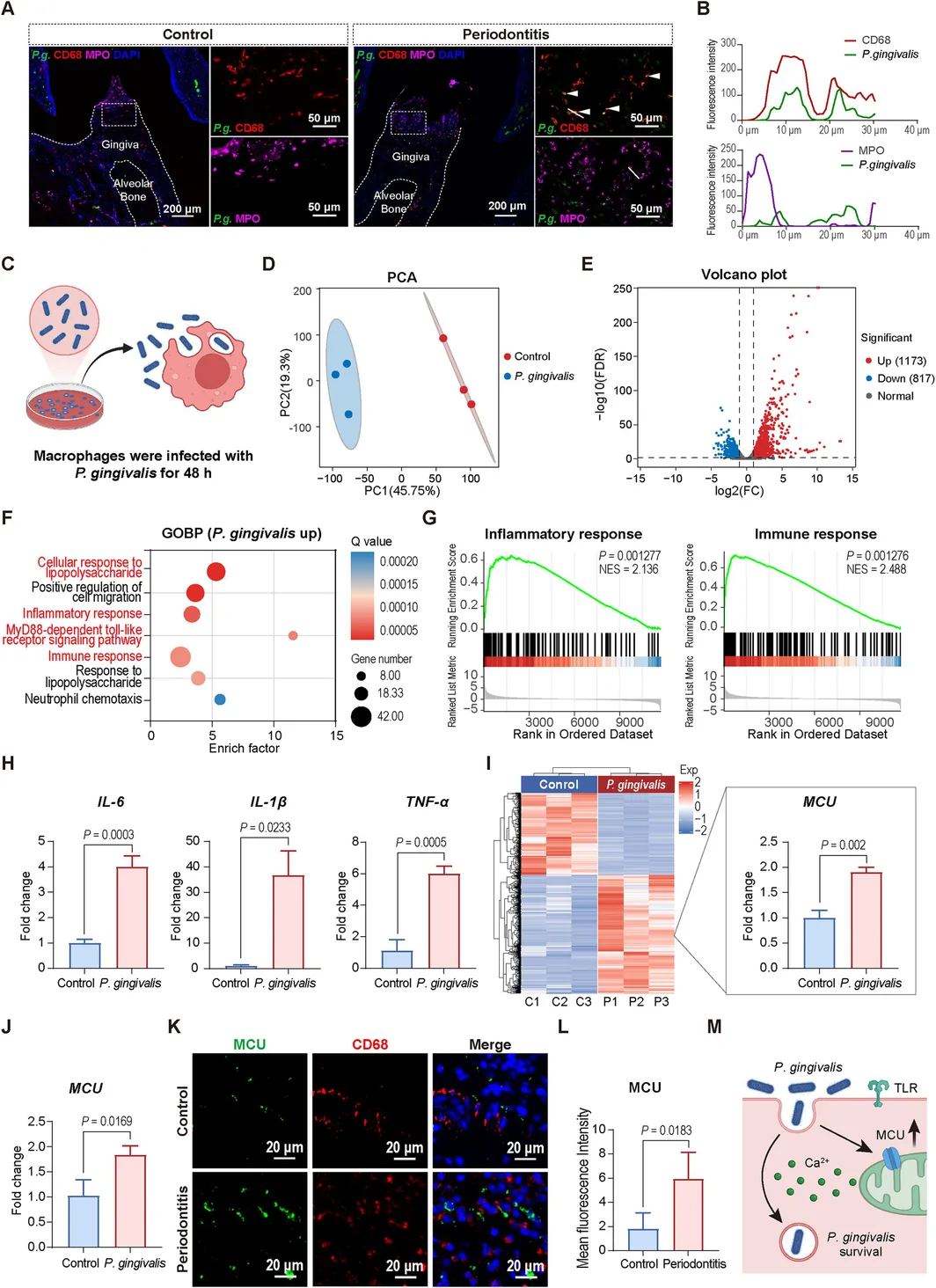
*P. gingivalis* infection drives an inflammatory response accompanied by upregulated MCU in macrophages. (A) Representative IF images depicting the colocalization of *P. gingivalis* (green), macrophages (CD68, red), and neutrophils (MPO, magenta) within periodontal tissues in control and periodontitis groups. White arrowheads indicate *P. gingivalis* ‐infected macrophages in periodontal tissues. (B) Fluorescence intensity profiles along the indicated lines in (A) showing the colocalization of *P. gingivalis* (green, *P. gingivalis* ) with CD68 (red, macrophage) and MPO (magenta, neutrophil) (C) Schematic illustration of the in vitro *P. gingivalis* ‐infected macrophages: Cells were infected with *P. gingivalis* (MOI = 100) for 4 h, washed, and cultured for total 48 h, with PBS‐treated cells serving as the control. (D) PCA plot of transcriptome data from the control and *P. gingivalis* ‐infected macrophages. (E) Volcano plot of the control and *P. gingivalis* ‐infected macrophages. (F) GO Biological Process analysis of upregulated pathways in the *P. gingivalis* ‐infected macrophages. (G) GSEA showing upregulation of inflammatory response and immune response in the *P. gingivalis* ‐infected macrophages. (H) qRT‐PCR analyses showing the *IL‐1β*, *IL‐6*, and *TNF‐α* gene expressions between control and *P. gingivalis* ‐infected macrophages. (I) Heatmap depicting DEGs, including MCU, between control and *P. gingivalis* ‐infected macrophages. (J) qRT‐PCR analyses showing the gene expression levels of *MCU* between control and *P. gingivalis* ‐infected macrophages. (K) Representative images depicting the MCU (green) expression in macrophages (CD68, red) within gingiva tissues dissected from the M2 at the mesial sites in control and periodontitis groups. (L) Quantification of the MFI of MCU. (M) Schematic diagram illustrating that *P. gingivalis* infection triggers a robust inflammatory response and upregulates the MCU expression in macrophages. Data are represented as mean ± SD. Student's t‐test was employed with equal variances, while Welch's t‐test was used for unequal variances (G, H, J). Created in BioRender. Deng, D. (2026) https://BioRender.com/j9wab3f; https://BioRender.com/65joamd.

After *P. gingivalis* infection, macrophages and neutrophils are major contributors to pathogen phagocytosis and clearance [15, 16, 18]. To determine which cell type is primarily responsible for *P. gingivalis* phagocytosis and clearance, we performed IF colocalization staining to detect the colocalization of *P. gingivalis* and potential phagocytotic cells (macrophages or neutrophils) by linear profiles of fluorescence intensity. The results showed that *P. gingivalis* was mainly colocalized with macrophages (CD68, red) as evidenced by the greater overlap between the fluorescence of the *P. gingivalis* and CD68, whereas no significant colocalization was detectable between the *P. gingivalis* and the neutrophils (MPO, magenta) (Figure 2A,B). These findings suggest that *P. gingivalis* in periodontal tissues was mainly phagocytosed into macrophages, indicating that macrophages are the major cells responsible for the phagocytosis and subsequent clearance of *P. gingivalis* .

However, the inability of macrophages to eliminate *P. gingivalis* resulted in local and systemic infection, thereby exacerbating periodontitis and its associated systemic diseases (Figure 1). To further explore the mechanisms by which *P. gingivalis* evades macrophage‐mediated clearance, macrophages were infected with *P. gingivalis* , and macrophages treated with PBS served as control group (Figure 2C). PCA showed that *P. gingivalis* ‐infected macrophage was separated from control group (Figure 2D), and volcano plot identified 1173 upregulated and 817 downregulated genes in *P. gingivalis* ‐infected macrophages (Figure 2E), indicating that *P. gingivalis* infection caused wide transcriptomic changes. GO biological process analysis revealed that the upregulated genes in *P. gingivalis* ‐infected macrophages were highly enriched in cellular responses to lipopolysaccharide (LPS), inflammatory responses, MyD88‐dependent toll‐like receptor signalling pathways, and immune responses (Figure 2F). GSEA further confirmed the upregulation of inflammatory and immune response in *P. gingivalis* ‐infected macrophages (Figure 2G). Consistently, qRT‐PCR analysis revealed the increased pro‐inflammatory *IL‐6*, *IL‐1β*, and *TNF‐α* gene expression in the *P. gingivalis* ‐infected macrophages (Figure 2H). ELISA analysis revealed the increased pro‐inflammatory cytokines IL‐1β and TNF‐α concentrations in the supernatant of *P. gingivalis* ‐infected macrophages (Figure S1). These results indicate that *P. gingivalis* triggered a robust inflammatory response upon phagocytosis by macrophages.

To identify key molecules in the phagocytosis and clearance of *P. gingivalis* , we then screened for differentially expressed genes (DEGs) (fold change ≥ 2; false discovery rate (FDR) < 0.05), and focused on genes related to bacterial phagocytosis and clearance. Calcium functions as a second messenger crucial for phagocytosis and macrophage‐mediated clearance [32, 33, 34]. Moreover, previous research has shown that MCU participated in regulating the intracellular calcium levels and phagocytic activity in macrophages [35]. Therefore, we specifically focused on the levels of MCU, and the heatmap showed that MCU was upregulated in *P. gingivalis* ‐infected macrophages (Figure 2I). These transcriptomic results suggested that *P. gingivalis* infection induced inflammatory response and MCU upregulation in macrophages. To further validate MCU upregulation in *P. gingivalis* ‐infected macrophages, we examined changes in *MCU* gene expression in vitro. qRT‐PCR analyses showed that the *MCU* was elevated in *P. gingivalis* ‐infected macrophages (Figure 2J). To verify the expression of MCU in macrophages from periodontal tissues in vivo, gingival tissues around M2 of control and periodontitis groups were extracted, and IF staining for macrophages (CD68) and MCU were performed. The gingiva IF staining results showed that the MCU expression was identified in macrophages and elevated in periodontitis group (Figure 2K,L). These findings indicate that *P. gingivalis* infection upregulates MCU in macrophages during periodontitis, suggesting potential role of MCU in aiding *P. gingivalis* survival and evasion of immune clearance (Figure 2M).

### 2.3 *P. gingivalis* Infection Induces Calcium Overload by Upregulating MCU Expression and Promotes Its Intracellular Survival in Macrophages

**FIGURE 3:**
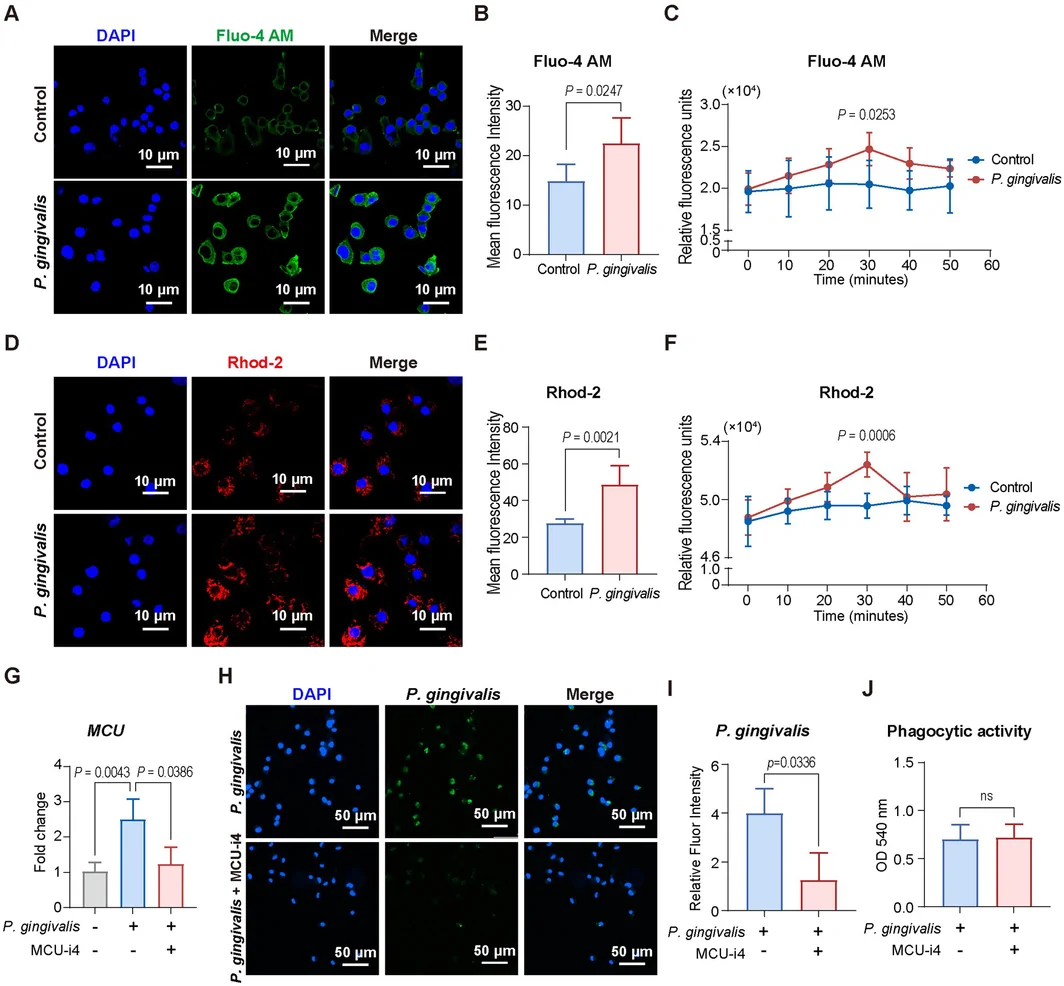
*P. gingivalis* infection impairs the intracellular bacteria clearance in macrophages through MCU‐mediated Ca^2+^ overload. (A) Representative fluorescence images showing the levels of the cytosolic Ca^2+^ (Fluo‐4 AM, green) in control and *P. gingivalis* ‐infected macrophages. (B) Quantification of Fluo‐4 AM MFI. (**C**) The changes of Fluo‐4 AM levels in control and *P. gingivalis* ‐infected macrophages over a 50‐min period. (D) Representative fluorescence images showing the levels of the mitochondrial Ca^2+^ (Rhod‐2, red) in control and *P. gingivalis* ‐infected macrophages. (E) Quantification of Rhod‐2 MFI. (F) The changes of Rhod‐2 AM levels in control and *P. gingivalis* ‐infected macrophages over a 50‐min period. (G) The *MCU* gene expression in macrophages between control, *P. gingivalis* , and *P. gingivalis* + MCU‐i4 (MCU inhibitor, 10 μM) group. (H) Representative IF images showing the intracellular survival of *P. gingivalis* in macrophages between *P. gingivalis* and *P. gingivalis* + MCU‐i4 groups. (**I**) Quantification of *P. gingivalis* MFI. (**J**) Quantification of phagocytic activity (OD 540 nm) in macrophages between *P. gingivalis* and *P. gingivalis* + MCU‐i4 groups. Data are represented as mean ± SD. Unpaired two‐tailed Student's t‐test was employed for comparisons between two groups (B, E, I, J). Two‐way ANOVA performed for experiments with two independent variables (C, F). One‐way ANOVA was used for analysing differences among three groups (G).

MCU is closely associated with both cytosolic and mitochondrial calcium levels [36, 37, 38], and calcium ions participate in bacterial phagocytosis and clearance [18, 20]. To determine whether increased MCU expression affects calcium homeostasis in macrophages after *P. gingivalis* infection, cytosolic calcium levels were measured using the calcium‐reactive fluorescence probe Fluo‐4 AM [39]. The *P. gingivalis* ‐infected macrophages exhibited a rise in cytosolic Ca^2+^ levels relative to the control group (Figure 3A,B). Dynamic monitoring over 50 min revealed that cytosolic Ca^2+^ in *P. gingivalis‐*infected macrophages gradually increased and reached a peak at 30 min, significantly surpassing the control group (Figure 3C). Subsequently, cytosolic Ca^2+^ levels of *P. gingivalis* ‐infected macrophages gradually declined but remained higher than that in the control group without reaching statistical significance. In addition, mitochondrial Ca^2+^ levels were measured using Rhod‐2 [40]. The results showed that mitochondrial Ca^2+^ (Rhod‐2 fluorescence) was also significantly elevated in *P. gingivalis* ‐infected macrophages (Figure 3D,E). Dynamic monitoring over 50 min similarly revealed that mitochondrial Ca^2+^ increased gradually after *P. gingivalis* infection, reached a peak at 30 min, significantly exceeding levels observed in the control group (Figure 3F), and then decreased. These results indicate that *P. gingivalis* infection increases MCU expression in macrophages, leading to elevated cytosolic and mitochondrial Ca^2+^ levels and ultimately causing calcium overload.

To clarify the role of MCU upregulation in *P. gingivalis* clearance, we utilized the MCU inhibitor MCU‐i4 to suppress MCU expression in *P. gingivalis* ‐infected macrophages (referred to as *P. gingivalis* + MCU‐i4 group). qRT‐PCR analyses showed that MCU‐i4 significantly reversed the overexpressed *MCU* in *P. gingivalis* ‐infected macrophages (Figure 3G). Meanwhile, the intracellular fluorescence intensity of *P. gingivalis* was reduced in the *P. gingivalis* + MCU‐i4 group compared to *P. gingivalis* ‐infected macrophages (Figure 3H,I), indicating that MCU inhibition either reduces *P. gingivalis* phagocytosis or enhances *P. gingivalis* clearance in macrophages. To further determine the mechanism underlying the reduction in *P. gingivalis* , we measured the phagocytic capacity of macrophages after MCU inhibition using the neutral red uptake assay. MCU inhibition did not alter the phagocytic capacity of macrophages (Figure 3J), indicating that the reduction in *P. gingivalis* is more likely attributable to enhanced intracellular clearance rather than reduced phagocytic capacity. These findings indicate that *P. gingivalis* infection induces MCU upregulation and calcium overload to promote its immune evasion, whereas MCU inhibition enhances the intracellular clearance of *P. gingivalis* .

### 2.4 Magnesium Enhances the *P. gingivalis* Clearance in Macrophages by Inhibiting the MCU Pathway and Reversing *P. gingivalis* ‐Induced Calcium Overload

**FIGURE 4:**
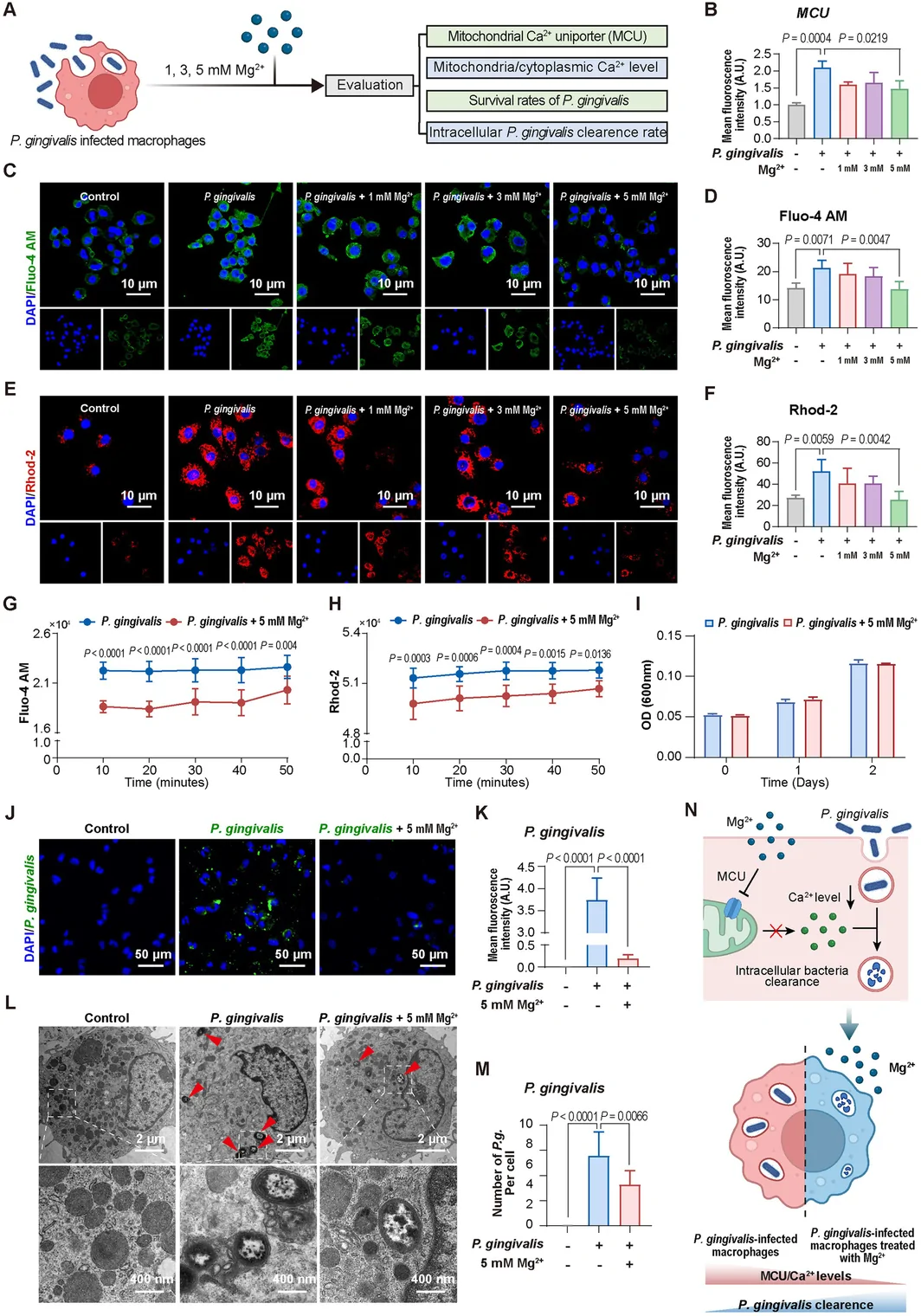
Magnesium enhances macrophages intracellular *P. gingivalis* clearance by suppressing the MCU pathway and restoring Ca^2+^ homeostasis. (A) Schematic representation of the experiment to investigate the magnesium‐mediated therapeutic effects on *P. gingivalis* ‐infected macrophages. *P. gingivalis* ‐infected macrophages were treated with 1, 3, and 5 mM Mg^2+^, resulting in five groups: Control, *P. gingivalis* , *P. gingivalis* + 1 mM Mg^2+^, *P. gingivalis* + 3 mM Mg^2+^, and *P. gingivalis* + 5 mM Mg^2+^. (B) qRT‐PCR analyses showing the gene expression of *MCU*. (C) Representative images showing the cytosolic Ca^2+^ levels (Fluo‐4 AM, green). (D) Quantification of Fluo‐4 AM MFI. (E) Representative images showing the mitochondrial Ca^2+^ levels (Rhod‐2, red). (F) Quantification of Rhod‐2 MFI. (G) The changes of Fluo‐4 AM levels in *P. gingivalis* and *P. gingivalis* + 5 mM Mg^2+^ groups over a 50‐min period. (H) The changes of Rhod‐2 AM levels in *P. gingivalis* and *P. gingivalis* + 5 mM Mg^2+^ groups over a 50‐min period. (I) The optical density (OD) values at 600 nm showing the bacterial growth inhibition of 5 mM Mg^2+^ on *P. gingivalis* over a 2‐day period. (J) Representative IF images depicting the effect of 5 mM Mg^2+^ on intracellular *P. gingivalis* clearance in macrophage. (K) Quantification of *P. gingivalis* MFI in control, *P. gingivalis* , and *P. gingivalis* + 5 mM Mg^2+^ groups. (L) Representative transmission electron microscopy images depicting the effect of 5 mM Mg^2+^ on intracellular clearance of *P. gingivalis* in macrophage. Red arrowheads indicate intracellular bacteria. (M) Quantification of the number of intracellular *P. gingivalis* per cell in control, *P. gingivalis* , and *P. gingivalis* + 5 mM Mg^2+^ groups. (N) Schematic illustrating the effect of Mg^2+^ on MCU, Ca^2+^ level, and *P. gingivalis* clearance in macrophages. Data are represented as mean ± SD. One‐way ANOVA was used for analysing differences among three or five groups (B, D, F, K, M). Two‐way ANOVA was performed for experiments with two independent variables (G, H, I). Created in BioRender. Deng, D. (2026) https://BioRender.com/ltfaaoo; https://biorender.com/emusx7c.

In addition to calcium overload, transcriptome analysis revealed that *P. gingivalis* infection significantly decreased the magnesium transporters CNNM1, CNNM2, and CNNM3 expression in macrophages (Figure S2A). Consistently, the concentration of Mg^2+^ was decreased in *P. gingivalis* ‐infected macrophages (Figure S2B). These results indicate that magnesium deficiency existed in *P. gingivalis* ‐infected macrophages. As magnesium is a natural calcium antagonist and participates in regulating calcium metabolism [41], we determine whether magnesium could reverse the upregulated MCU expression and calcium overload in *P. gingivalis* ‐infected macrophages. To verify this hypothesis, *P. gingivalis* ‐infected macrophages were treated with graded concentrations of MgCl_2_ (1, 3, and 5 mM), and termed as 1, 3, and 5 mM Mg^2+^ (Figure 4A). The results showed that 5 mM Mg^2+^ could significantly reverse the *P. gingivalis* ‐induced upregulated *MCU* gene expression (Figure 4B), whereas 1 mM and 3 mM Mg^2+^ showed a negative effect without statistical significance. Similarly, Fluo‐4 AM staining results showed that only 5 mM Mg^2+^ significantly reduced *P. gingivalis* ‐induced cytosolic calcium overload (Figure 4C,D). Further mitochondrial calcium measurements using Rhod‐2 showed that only 5 mM Mg^2+^ reduced mitochondrial Ca^2+^ levels (Figure 4E,F). These findings indicate that the inhibition of calcium overload was most evident at an Mg^2+^ concentration of 5 mM, and 5 mM Mg^2+^ was applied in subsequent experiments. Dynamic monitoring further confirmed that 5 mM Mg^2+^ could sustainably reduce both cytosolic and mitochondrial calcium overload induced by *P. gingivalis* infection (Figure 4G,H).

To determine whether restoration of calcium homeostasis by magnesium could enhance macrophage‐mediated *P. gingivalis* clearance, we evaluated the effect of magnesium on intracellular *P. gingivalis* . First, we examined whether magnesium could directly inhibit *P. gingivalis* growth. During the 2‐day treatment period, 5 mM Mg^2+^ did not significantly affect *P. gingivalis* growth (Figure 4I), indicating that magnesium does not inhibit *P. gingivalis* growth. Notably, IF staining revealed that fluorescence intensity of intracellular *P. gingivalis* was significantly decreased by 5 mM Mg^2+^ in macrophages (Figure 4J,K), and representative transmission electron microscopy (TEM) images confirmed that 5 mM Mg^2+^ significantly reduced the number of intracellular *P. gingivalis* in macrophages (Figure 4L,M). These findings suggest that magnesium does not directly inhibit *P. gingivalis* growth but instead enhances macrophage‐mediated clearance of the bacteria. Overall, these results suggest that magnesium reduces MCU upregulation, reverses calcium overload in *P. gingivalis* ‐infected macrophages, and thereby enhances the macrophage‐mediated clearance of *P. gingivalis* (Figure 4N).

### 2.5 Magnesium Effectively Inhibited Both the Local and Systemic Infection of *P. gingivalis* and Alleviates Inflammatory Response in Periodontitis Rat

**FIGURE 5:**
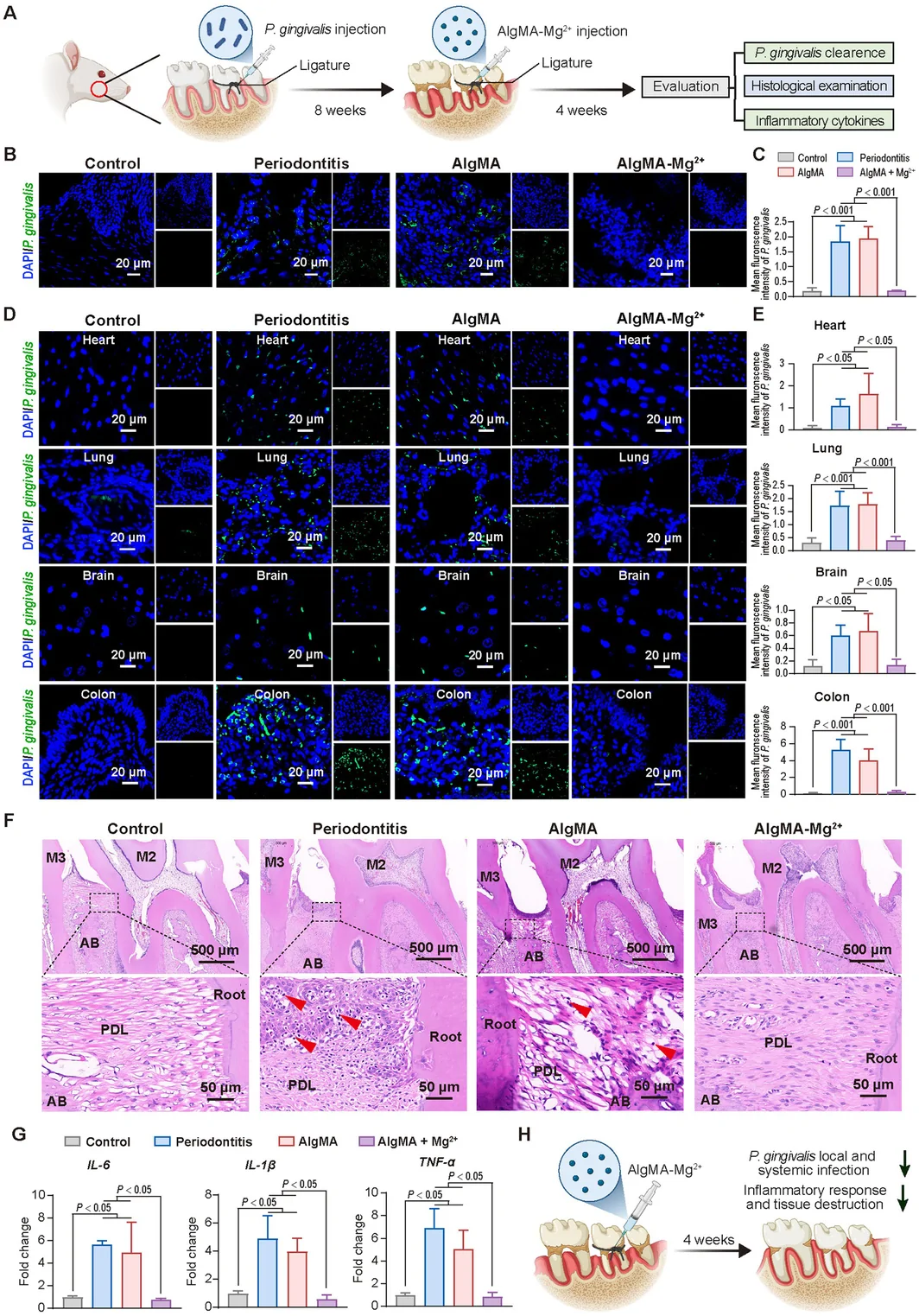
Local administration of magnesium enhances local and systemic clearance of *P. gingivalis* and alleviates inflammation and tissue destruction in periodontitis. (A) Schematic representation of the experiment for the in vivo study. A rat model of periodontitis was established and injected with alginate methacryloyl (AlgMA) or Mg^2+^‐loaded AlgMA (AlgMA‐Mg^2+^) hydrogels, resulting in four groups: Control, periodontitis, AlgMA, and AlgMA‐Mg^2+^. (B) Representative images showing the effect of Mg^2+^ on *P. gingivalis* ‐related signals within gingiva tissues. (**C**) Quantification of the *P. gingivalis* MFI. (D) Representative images showing the effect of Mg^2+^ on *P. gingivalis* ‐related signals within systemic organs including heart, lung, brain, and colon. (E) Quantification of the *P. gingivalis* MFI. (F) Representative H&E staining of periodontal tissue depicting the anti‐inflammatory effect of Mg^2+^ on periodontitis. Red arrowheads indicate the inflammatory cells. Periodontal ligament (PDL). Alveolar bone (AB). Third molar (M3). (G) The *IL‐6, IL‐1β*, and *TNF‐α* gene expression of gingiva tissues dissected from M2 at the mesial sites. (H) Schematic diagram illustrating the therapeutic effect of the AlgMA‐Mg^2+^ hydrogel on the local and systemic clearance of *P. gingivalis* and the alleviation of inflammation and tissue destruction in periodontitis. Data are represented as mean ± SD. One‐way ANOVA was used for analysing differences among groups with normal distribution, while Kruskal‐Wallis test was used with non‐normal distribution (C, E, G). Created in BioRender. Deng, D. (2026) https://BioRender.com/a0ykzs2; https://BioRender.com/5epcw2b.

To evaluate the effect of magnesium on *P. gingivalis* clearance in vivo, a periodontitis rat model was established, and Mg^2+^ was locally administered. As periodontitis is a chronic inflammatory and destructive disease [42, 43], direct injection of Mg^2+^ may lead to burst release and limited therapeutic efficacy. Therefore, AlgMA hydrogel was used as a carrier to sustainably deliver Mg^2+^ due to its good biocompatibility and sustained drug release properties [44, 45]. AlgMA‐Mg^2+^ hydrogel containing 7.5 mM Mg^2+^ could continuously release Mg^2+^ for up to 35 days, with a cumulative release rate of 26.5% on Day 3 (Figure S3), indicating that the Mg^2+^ concentration could be maintained above the effective level (5 mM) (Figure 4) during the dosing interval of 3 days. After the establishment of the periodontitis model, AlgMA‐Mg^2+^ hydrogel was injected into the gingival sulcus of M2 (termed as AlgMA‐Mg^2+^ group) every 3 days, while AlgMA alone served as the vehicle control (termed as AlgMA group), to assess whether Mg^2+^ could enhance immune clearance of *P. gingivalis* and alleviate periodontitis in vivo (Figure 5A). The IF staining depicted that *P. gingivalis* ‐related signals were increased within gingiva tissues in both periodontitis and AlgMA group, while these signals were decreased in AlgMA‐Mg^2+^ group (Figure 5B,C), indicating that magnesium effectively reduced the local accumulation of *P. gingivalis* or its related components in periodontal tissue.

To further evaluate the effect of magnesium on systemic dissemination of *P. gingivalis* , we examined the *P. gingivalis* ‐related signals in distant organs (heart, lung, brain, and colon) after AlgMA‐Mg^2+^ treatment. IF staining depicted that *P. gingivalis* ‐related signals were increased within the heart, lung, brain, and colon tissues in both periodontitis and AlgMA groups, whereas these signals were decreased in the AlgMA‐Mg^2+^ group (Figure 5D,E). These findings indicate that magnesium not only decreases the local presence of *P. gingivalis* ‐related components in the periodontium, but also reduces systemic *P. gingivalis* dissemination.

To determine whether magnesium‐mediated clearance of *P. gingivalis* could alleviate periodontal inflammation and bone loss, H&E staining of periodontal tissues was performed, and inflammatory cytokine gene expression of gingival tissues was measured respectively. H&E staining showed that both the periodontitis and AlgMA groups exhibited extensive inflammatory cell infiltration and PDL and alveolar bone (AB) destruction (Figure 5F), accompanied by increased *IL‐6, IL‐1β*, and *TNF‐α* gene expression of gingiva (Figure 5G). In contrast, AlgMA‐Mg^2+^ significantly decreased inflammatory cell infiltration, periodontal destruction, and *IL‐6, IL‐1β*, and *TNF‐α* gene expression (Figure 5F,G). Masson's trichrome staining demonstrated that the PDL fibres in the periodontitis and AlgMA treatment group were irregularly arranged and immature (blue). In contrast, the PDL fibres in the AlgMA‐Mg^2+^ treatment group were regularly arranged and mature (red), indicating better regeneration of the PDL fibres (Figure S4). These findings indicate that magnesium promotes the *P. gingivalis* clearance and alleviates periodontal inflammation and tissue destruction (Figure 5H).

## 3 Discussion

**FIGURE 6:**
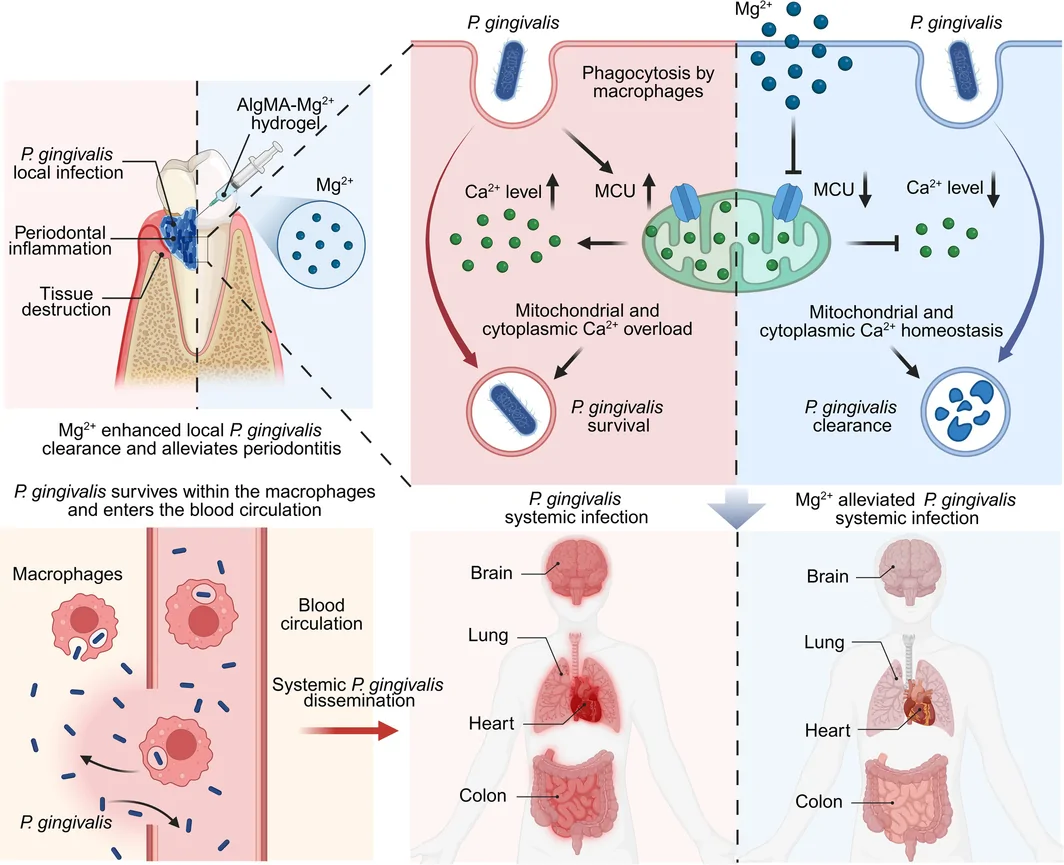
Magnesium restores macrophage clearance of *P. gingivalis* by inhibiting MCU‐mediated calcium overload. *P. gingivalis* is mainly phagocytosed by macrophages but escapes intracellular clearance via MCU‐mediated mitochondrial and cytosolic Ca^2+^ overload in periodontitis. The persistent *P. gingivalis* infection leads to local periodontal inflammation and tissue destruction, and promotes *P. gingivalis* dissemination to distant organs through the blood circulation. As a natural antagonist of Ca^2+^, magnesium treatment suppresses overexpressed MCU, restores Ca^2+^ homeostasis, enhances macrophage‐mediated *P. gingivalis* clearance, and thereby reduces local and systemic *P. gingivalis* infection as well as periodontal tissue damage. Created in BioRender. Deng, D. (2026) https://BioRender.com/pmz4i1c.

In this study, we identified MCU‐mediated calcium overload as an unrecognized mechanism by which *P. gingivalis* evaded macrophage‐mediated clearance in periodontitis. *P. gingivalis* could upregulate MCU expression, leading to cytosolic and mitochondrial Ca^2+^ overload, which impairs intracellular clearance capacity of macrophages. The persistent local *P. gingivalis* infection led to periodontal tissue destruction and systemic dissemination. Notably, magnesium treatment suppressed MCU, restored Ca^2+^ homeostasis, enhanced macrophage clearance of *P. gingivalis* , and reduced both local periodontal and systemic infection, thereby attenuating periodontal tissue destruction. These findings highlight magnesium as a promising host‐directed immunomodulatory therapy for periodontitis and its associated systemic diseases (Figure 6).

Mounting evidence has demonstrated that *P. gingivalis* can evade host immune clearance and persist within the human host, thereby promoting the periodontitis progression and increasing the risk of its related systemic disease [46]. Previous studies reported that *P. gingivalis* can invade epithelial, endothelial, dendritic cells, and macrophages, where it interferes with critical antimicrobial pathways [14, 17, 47, 48]. For instance, *P. gingivalis* can inhibit autophagosome‐lysosome fusion and autophagic flux in oral epithelial cells by modulating lysosome efflux, enhancing its intracellular survival [17]. In neutrophils, *P. gingivalis* could also reduce the bactericidal activity through interactions with CD47 and TSP‐1, enabling its survival in an inflammatory environment [15]. Moreover, the virulence factors of *P. gingivalis* itself could directly suppress host immune clearance. For instance, gingipain from *P. gingivalis* can hydrolyze CD14 and disrupt the CD14‐mediated Vav‐Rac/Cdc42 signalling cascade, thereby suppressing macrophage phagocytosis and intracellular bacterial clearance [16], and inhibition of gingipains could enhance macrophage‐mediated bacterial phagocytosis and clearance [6]. Moreover, the sialidase of *P. gingivalis* further promotes its immune evasion by regulating macrophage polarization and phagocytosis [49]. These observations are consistent with our results and further support our notion that *P. gingivalis* caused periodontal and systemic infection (Figure 1) by evading macrophage‐mediated clearance (Figure 2A), emphasizing the need to understand how *P. gingivalis* escapes immune clearance in macrophages. Building upon previous findings, our study further proposes that MCU‐mediated mitochondrial Ca^2+^ overload serves as another mechanism underlying the impaired intracellular clearance of *P. gingivalis* in macrophages.

Ca^2+^ signalling is crucial for the normal antibacterial functions of macrophages, including phagocytosis, phagosome maturation, lysosomal activity, and intracellular bacterial killing [21, 22, 23]. As the primary channel mediating mitochondrial Ca^2+^ uptake, MCU is involved in regulating phagocytic activity, making it essential for maintaining normal macrophage functions [50, 51]. On the one hand, inhibition of MCU can impair the phagocytic capacity of macrophages [35]. On the other hand, excessive activation of MCU leads to mitochondrial calcium overload, thereby triggering a cascade of cellular dysfunctions and pathological changes [52]. For example, inhibition of MCU can restore mitochondrial dysfunction and reduce inflammatory responses in macrophages [53]. Therefore, while MCU is essential for normal macrophage phagocytosis, its overactivation or over inhibition could induce macrophage dysfunction. In our study, we found that *P. gingivalis* infection upregulated MCU expression, leading to increased calcium levels in both the mitochondria and cytosol, indicating that *P. gingivalis* induces a pathological state of calcium overload in macrophages through the MCU pathway (Figures 2G–K and 3A–F). It should be emphasized that our findings do not imply that elevated Ca^2+^ signalling is inherently detrimental, but rather revealed that calcium dysregulation is one of the pathological processes of *P. gingivalis* infection, especially the excessive accumulation of mitochondrial Ca^2+^ mediated by MCU. These observations reveal a previously unrecognized link between *P. gingivalis* infection and MCU‐mediated calcium signalling dysregulation in macrophages during periodontitis.

Subsequently, our data reveal that MCU‐mediated calcium overload is a key mechanism underlying the impaired *P. gingivalis* clearance in macrophages. Inhibition of MCU could significantly reduce the number of intracellular *P. gingivalis* without affecting the phagocytic capacity of macrophages (Figure 3G–J), suggesting that the post‐phagocytic intracellular *P. gingivalis* clearance capacity was impaired. Although a previous study reported that MCU inhibition impaired macrophage phagocytosis under *E. coli* infection [35], the discrepancy may be explained by differences in bacterial species, infection models, and pathological contexts.

Mechanistically, Ca^2+^ is essential for autophagosome formation and autophagosome–lysosome fusion [54, 55]. Given that *P. gingivalis* has been reported to disrupt autophagosome–lysosome fusion and promote its intracellular survival [15, 17, 56], MCU‐mediated mitochondrial calcium overload is more likely to impair intracellular bacteria clearance by interfering with the autophagolysosomal function. These findings indicate that *P. gingivalis* employs multiple strategies to escape from immune clearance, and our study adds MCU‐mediated calcium overload as another important component of *P. gingivalis* immune evasion. Considering that other Gram‐negative bacteria or their toxic component LPS may also enhance Ca^2+^ signalling [57, 58], we propose that MCU‐mediated calcium overload may represent a broadly applicable bacterial immune evasion strategy.

Another significant finding is the identification of magnesium as a protective agent of MCU‐mediated calcium overload. Compared to direct MCU inhibition using MCU‐i4, magnesium may have greater translational potential because it is a naturally occurring ion in the body with good biocompatibility and known clinical safety [59, 60, 61]. In contrast, pharmacological MCU inhibitors are currently used mainly as experimental research tools, and their long‐term in vivo safety and potential impacts on physiological Ca^2+^ homeostasis require further elucidation. Our transcriptomic analysis revealed that several magnesium transporters, including CNNM1, CNNM2, and CNNM3 [62], were downregulated in *P. gingivalis* ‐infected macrophages (Figure S2), suggesting that the intracellular levels of magnesium were significantly decreased. Therefore, we supplemented *P. gingivalis* ‐infected macrophages with exogenous magnesium. This treatment successfully reduced MCU expression and reversed both cytosolic and mitochondrial Ca^2+^ overload, with 5 mM showing the strongest effect (Figure 4B–F). Notably, magnesium did not directly inhibit *P. gingivalis* growth, but rather enhance the intracellular bacterial clearance in macrophages (Figure 4I–M). These data indicate that magnesium could promote *P. gingivalis* clearance through restoration of host immune clearance rather than direct antibacterial effects. Since conventional antibacterial agents are prone to inducing antibacterial resistance [63, 64], the magnesium‐based host‐directed immunomodulatory therapy is appealing in periodontitis and other infectious diseases.

In addition to restoring MCU‐mediated Ca^2+^ overload, magnesium may also regulate macrophage function through other mechanisms. Previous studies have shown that Mg^2+^ can influence mitochondrial homeostasis, metabolic activity, inflammatory signalling, inflammasome activation, and macrophage polarization [65, 66, 67, 68]. Improved mitochondrial function and remodelling of macrophage immune phenotypes may also collectively contribute to bacterial clearance. Therefore, the protective effects of magnesium observed in our study were not entirely dependent on the MCU‐Ca^2+^ axis, and we do not exclude the involvement of other magnesium‐regulated pathways.

Our in vivo findings further confirm the translational potential of magnesium. AlgMA hydrogel possesses good biocompatibility and controllable drug release properties and has been utilized to promote periodontal regeneration [69, 70, 71]. Therefore, AlgMA was employed as a delivery carrier for magnesium in this study. AlgMA‐Mg^2+^ not only inhibited the local *P. gingivalis* infection in periodontal tissues but also prevented its systemic spread (Figure 5B–E). These results suggest that the magnesium‐mediated enhancement of bacterial clearance by local macrophages likely reduces the periodontal *P. gingivalis* burden, thereby limiting the dissemination of the bacteria or their components to distant organs. However, based on the current data, we cannot entirely rule out the possibility that locally released magnesium enters the systemic circulation and exerts direct systemic effects. Additionally, AlgMA‐Mg^2+^ treatment attenuated the periodontal inflammation and tissue destruction (Figure 5F,G). Together, these results indicated that magnesium‐based host‐directed immunomodulatory therapy simultaneously eliminated bacteria, alleviated inflammation, and promoted tissue repair, breaking the vicious cycle of infection‐inflammation in periodontitis [72], and offering new insights for periodontitis and other chronic inflammatory diseases.

Several limitations should be acknowledged. While our study establishes the upregulation of MCU and calcium overload as key events in *P. gingivalis* immune evasion, the specific *P. gingivalis* virulence factor responsible for MCU upregulation remains to be elucidated. Future investigations are warranted to determine whether virulence factors such as gingipains, LPS, or fimbriae directly regulate MCU expression [16]. Second, although our data support the conclusion that magnesium enhances intracellular bacterial clearance, its downstream mechanisms have not been fully elucidated. Further research is required to investigate whether magnesium enhances bacterial clearance mediated by autophagy or other pathways [17, 73].

In conclusion, our study identifies the MCU‐mediated calcium overload in macrophage as a previously unrecognized mechanism of *P. gingivalis* immune evasion. Moreover, magnesium reverses *P. gingivalis* ‐induced calcium overload, promotes macrophage‐mediated local and systemic *P. gingivalis* clearance, and alleviates inflammation and tissue destruction in periodontitis. This work enhances our comprehension of periodontal pathogenesis and provides practical insights for developing novel host‐directed immunomodulatory therapy to treat periodontitis and reduce its associated systemic health risks.

## 4 Materials and Methods

### 4.1 Cell Culture

THP‐1 monocytes (TIB‐202, ATCC, VA, USA) were maintained in RPMI 1640 medium (C11875500BT, Gibco, NY, USA) with 10% fetal bovine serum (FBS) (A5256701, Gibco) at 37°C in a 5% CO_2_ humidified incubator. For monocyte–macrophage differentiation, THP‐1 cells were treated with 200 ng/mL PMA (HY‐18739, MCE, NJ, USA) for 48 h.

### 4.2 *P. gingivalis* Culture and Infection of Macrophage

All in vitro and in vivo experiments in this study were performed using the *P. gingivalis* ATCC 33277 strain. This strain was purchased from BIO SCI Biotechnology (Zhejiang, China), and maintained in brain heart infusion (BHI) broth (HB8297‐1, Hopebio, Qingdao, China) containing 40% BHI, 0.5% hemin chloride (HB0310a, Hopebio), 0.1% vitamin K_1_ (HB8462a, Hopebio), and 20% defibrinated sheep blood (TX0030, Solarbio, Beijing, China). Bacteria were maintained anaerobically at 37°C in 10% H_2_, 10% CO_2_, and 80% N_2_ using a gas pack system (AN0025A, Thermo, CA, USA). For infection experiments, PMA‐differentiated macrophages were infected with *P. gingivalis* at a 100 MOI for 4 h [8, 17]. After infection, the medium was substituted with medium containing 500 μg/mL gentamicin (L1312, Solarbio) for 30 min to eliminate extracellular bacteria [8].

### 4.3 Transcriptome Sequencing and Analysis

#### 4.3.1 RNA Extraction, Library Construction, and Sequencing

RNA was extracted from *P. gingivalis* ‐infected and uninfected control macrophages using TRIzol (15596018CN, Thermo). The NanoDrop 2000 spectrophotometer was used to measure RNA concentration and purity, and the RNA Nano 6000 Assay Kit on an Agilent Bioanalyzer 2100 was used to assess RNA integrity. Libraries were constructed and sequenced on the Illumina NovaSeq platform with 150 bp read lengths.

#### 4.3.2 Transcriptome Data Analysis

Differential expression analysis was performed using the DESeq2 R package. The resulting *p*‐values were adjusted for multiple testing using the Benjamini‐Hochberg method to control the FDR. Genes with adjusted *p*‐value (*Q*‐value) < 0.05, fold change ≥ 2, and FDR < 0.05 were considered DEGs. Gene function was annotated using GO and KEGG databases. The clusterProfiler R package was utilized to conduct GO enrichment analysis on DEGs.

### 4.4 qRT‐PCR Analysis of MCU

RNA was extracted using TRIzol (MI00617, Mishushengwu, Shaanxi, China). cDNA was synthesized using reverse transcription kit (11141ES60, Yeasen, Shanghai, China). qPCR was performed using a SYBR Green qPCR mix (11201ES03, Yeasen) on Bio‐Rad CFX96 qPCR instrument (Bio‐Rad, CA, USA). The primer sequences were as follows:

*MCU* (Forward): 5′‐ATTGCTGCTGCTGCTGCTACTC‐3′.

*MCU* (Reverse): 5′‐GGGTGGTGGAGGGAGAAGGAAG‐3′.

*Actin* (Forward): 5′‐TGGCACCCAGCACAATGAA‐3′.

*Actin* (Reverse): 5′‐CTAAGTCATAGTCCGCCTAGAAGCA‐3′.

### 4.5 Elisa

Culture supernatants were collected from the control and *P. gingivalis* ‐infected macrophages after an additional 24‐h incubation. The collected supernatants were centrifuged to remove cell debris and then stored at −80°C until analysis. The concentrations of IL‐1β and TNF‐α were detected using the IL‐1β ELISA kit (97028ES96, Yeasen, Shanghai, China) and TNF‐α ELISA kit (97072ES48, Yeasen, Shanghai, China).

### 4.6 Fluorescence Imaging of *P. gingivalis* Infection

Macrophages were fixed and incubated with anti‐ *P. gingivalis* (1:300; D376‐3, MBL International, MA, USA) overnight at 4°C. Then, cells were treated with Alexa Fluor 488 (1:400; GB25303, Servicebio, Hubei, China) for 1 h at room temperature. Nuclei were counterstained with DAPI (10 μM; C1002, Beyotime, Shanghai, China) for 5 mins. Images were captured using a confocal laser scanning microscope (Nikon, Tokyo, Japan), and fluorescence intensity was measured using ImageJ 1.53k.

### 4.7 Measurement of Cytosolic and Mitochondrial Calcium Levels

Fluo‐4 AM and Rhod‐2 were utilized to measure cytosolic and mitochondrial Ca^2+^ levels, respectively. Macrophages were infected by *P. gingivalis* as previously described and then treated with PBS (control), MCU‐i4 (10 μM; HY‐138620, MedChemExpress), or different concentrations of Mg^2+^ (1, 3, 5 mM MgCl_2_; C0221G, Beyotime) for 48 h [74, 75]. Post treatment, extracellular bacteria were eliminated by gentamicin. Cells were incubated in fresh RPMI 1640 for 30 min and then loaded with Fluo‐4 AM or Rhod‐2 working solution for 30 min. Ca^2+^ levels were then visualized using a confocal microscope, with excitation wavelengths of 488 nm (Fluo‐4 AM) and 561 nm (Rhod‐2), and quantified using ImageJ 1.53k. In addition, dynamic changes in fluorescence level of the sample were then further monitored using Microplate Reader (Spark, Tecan, Männedorf, Switzerland). Fluorescence values were recorded every 10 min from 10 to 60 min. The excitation/emission wavelengths were set at 494/516 nm for Fluo‐4 AM and 549/578 nm for Rhod‐2.

### 4.8 Measurement of the Intracellular Mg^2+^ Concentration

Intracellular magnesium levels in macrophages were quantified using a colorimetric Magnesium Assay Kit (BC8337, Solarbio, Beijing, China) in accordance with the manufacturer's protocol. Briefly, following *P. gingivalis* infection or PBS (control) treatments, macrophages were harvested, washed with PBS, and lysed. The cell lysates were then centrifuged to remove cellular debris, and the supernatants were collected for the assay of Mg^2+^ concentration.

### 4.9 *P. gingivalis* Growth Rate Assay

A total of 1 × 10^5^ *P. gingivalis* was cultured in a 96‐well plate for 24 h. The *P. gingivalis* were then treated with either PBS or 5 mM Mg^2+^ and incubated for an additional 48 h. Optical density at 600 nm was recorded to determine the bacterial concentration and assess the growth‐inhibitory effect of 5 mM Mg^2+^ [76, 77].

### 4.10 Macrophage Phagocytosis Assay

Macrophage phagocytic capacity was assessed with a Neutral Red Uptake Assay Kit (C0013, Beyotime) [78, 79]. A total of 5 × 10^4^ cells were plated in a 96‐well plate for 24 h. Post *P. gingivalis* infection and PBS or MCU‐i4 treatment, 20 μL of neutral red stain was added per well, followed by a 2‐h incubation. Subsequently, 200 μL of Neutral Red assay lysis solution was added per well, followed by shaking for 10 min. Absorbance at 540 nm was recorded.

### 4.11 Animal Experiments

#### 4.11.1 Ethics Statement

Animal experiments were approved by the Animal Research Ethics Committee of the Fourth Military Medical University (Approval no.: 20260036) and followed the NIH Guide for the Care and Use of Laboratory Animals (NIH, MD, USA).

#### 4.11.2 Preparation and Measurement of Mg^2+^ Release Profile of AlgMA/AlgMA‐Mg^2+^ Hydrogel

Hydrogel precursor solutions were prepared as previously reported [45, 80]. AlgMA (EFL‐AlgMA‐50 K, Engineering for life, Zhejiang, China) was dissolved in ddwater to 2% (w/v), with 0.5% (w/v) lithium acylphosphinate added as the photoinitiator. Mg^2+^ (MgCl_2_) was incorporated into the precursor solution to a final concentration of 7.5 mM [75], forming AlgMA‐Mg^2+^ hydrogel. AlgMA or AlgMA‐Mg^2+^ hydrogels were cross‐linked with 405 nm visible blue light for 15 s.

To evaluate the release profile of Mg^2+^ from the AlgMA‐Mg^2+^ hydrogel, the prepared AlgMA‐Mg^2+^ hydrogels (initially containing 7.5 mM Mg^2+^) were immersed in PBS and incubated at 37°C. The supernatant was collected at Day 1, 3, 5, 7, 14, 28, and 35 to measure the released Mg^2+^, and an equal volume of fresh PBS was supplemented [81]. The concentration of Mg^2+^ in the collected supernatants was quantified using a colorimetric Magnesium Assay Kit (BC8337, Solarbio, Beijing, China) according to the manufacturer's instructions, and the cumulative release rate of Mg^2+^ was calculated.

#### 4.11.3 Establishment of Experimental Periodontitis Rat Models and Treatment Groups

8‐week‐old male SD rats were obtained from the Animal Center of the Fourth Military Medical University. Experimental periodontitis was induced by placing sterile 3–0 silk ligature around the bilateral maxillary M2. One day post‐ligation, 2 × 10^8^ *P. gingivalis* was administered to the buccal and lingual sides every other day [8, 14, 42]. After 8 weeks, the periodontitis group was randomly assigned to receive injections of 30 μL PBS, alginate hydrogel (Alg) or Mg^2+^‐loaded alginate hydrogel (AlgMA‐Mg^2+^), resulting in four groups: (1) control (healthy); (2) periodontitis (periodontitis + PBS); (3) AlgMA (periodontitis + AlgMA); (4) AlgMA‐Mg^2+^ (periodontitis + AlgMA‐Mg^2+^). Each mouse received an injection every 3 days. After 4 weeks, the rats' maxillae and major organs (heart, brain, lung, and colon) were fixed in 4% paraformaldehyde.

### 4.12 Micro‐CT Analysis

Maxillae were scanned using a micro‐CT system (AX2000, AlwaysImaging, Shanghai, China) at a 7 μm resolution. Data reconstruction and analysis were performed using VGstudioMAX 3.5.1. Three‐dimensional data was utilized to assess new bone growth in a region of interest (ROI). ROI was identified as the tissue around the M2, excluding the tooth roots. Each ROI was examined to measure CEJ, BV/TV, and Tb.Sp.

### 4.13 Histological Analysis

Maxillae specimens were decalcified using EDTA and embedded in paraffin after formalin fixation. Specimens were sectioned and stained with H&E and Masson to observe histological changes as previously described [81].

### 4.14 IF Analysis of Local Bacteria Infection and Co‐Localization

To examine the co‐localization of *P. gingivalis* , macrophages, and neutrophils in periodontal tissue adjacent to the M2, sections were processed using a tyramide‐based signal amplification (TSA) kit. Primary antibodies were used to incubate sections overnight at 4°C followed by a one‐hour incubation with an HRP‐linked secondary antibody and a 10‐min treatment with a TSA. The following primary antibodies were utilized: anti‐CD68 (1:2000; GB153109, Servicebio), anti‐MPO (1:5000; GB150006, Servicebio), and anti‐ *P. gingivalis* (1:500; D376‐3, MBL International). The detection system included HRP‐linked secondary antibody (1:500; GB23303; GB23301, Servicebio) and TSA kits with IF488 (1:500; G1231, Servicebio), IF555 (1:500; G1233, Servicebio), and IF647 (1:500; G1232, Servicebio). The colocalization signals indicated by the white line were analysed using the ImageJ 1.53k.

### 4.15 IF Analysis of MCU Expression in Macrophages Within Gingiva Tissues

To evaluate the MCU expression of macrophages in gingival tissues adjacent to the M2, sections were processed as described above. The following primary antibodies were utilized: anti‐CD68 (28058‐1‐AP, 1:500, Proteintech, Hubei, China) and anti‐MCU (D2Z3B, 10 μM, CST, Danvers, MA, USA), followed by HRP and TSA kits with IF488 (1:500; G1231, Servicebio) and IF555 (1:500; G1233, Servicebio).

### 4.16 IF Analysis of Systemic *P. gingivalis* Infection

Heart, lung, brain, and intestine tissues were collected, fixed, and paraffin‐embedded. Sections were treated with anti‐ *P. gingivalis* antibody (D376‐3, 1:500, MBL International), followed by Alexa Fluor 488 (GB25301, 1:400, Servicebio), and counterstained with DAPI. Images were captured with a Pannoramic MIDI scanner (3DHISTECH, Budapest, Hungray).

### 4.17 qRT‐PCR Analysis

Gingival tissues were homogenized in TRIzol reagent (MI00617, Mishushengwu) for RNA extraction. For in vitro experiments, human‐specific primers were used. For rat gingival tissues, rat‐specific primers were used. The primer sequences were as follows:

Rat‐*TNF‐α* (Forward): 5′‐ATGGGCTCCCTCTCATCAGT‐3′.

Rat‐*TNF‐α* (Reverse): 5′‐GCTTGGTGGTTTGCTACGAC‐3′.

Rat‐*IL‐1β* (Forward): 5′‐AGCTATGGCAACTGTCCCTG‐3′.

Rat‐*IL‐1β* (Reverse): 5′‐GGGCTTGGAAGCAATCCTTAATC‐3′.

Rat‐*IL‐6* (Forward): 5′‐GCCCACCAGGAACGAAAGTC‐3′.

Rat‐*IL‐6* (Reverse): 5′‐TGGCTGGAAGTCTCTTGCGG‐3′.

Rat‐*Actin* (Forward): 5′‐CCCATCTATGAGGGTTACGC‐3′.

Rat‐*Actin* (Reverse): 5′‐TTTAATGTCACGCACGATTTC‐3′.

Human‐*TNF‐α* (Forward): 5′‐GCCCATGTTGTAGCAAACCC‐3′.

Human‐*TNF‐α* (Reverse): 5′‐TGAGGTACAGGCCCTCTGAT‐3′.

Human‐*IL‐1β* (Forward): 5′‐CAGAAGTACCTGAGCTCGCC‐3′.

Human‐*IL‐1β* (Reverse): 5′‐CCTGGAAGGAGCACTTCATCT‐3′.

Human‐*IL‐6* (Forward): 5′‐CCACCGGGAACGAAAGAGAA‐3′.

Human‐*IL‐6* (Reverse): 5′‐GAGAAGGCAACTGGACCGAA‐3′.

Human‐*Actin* (Forward): 5′‐TGGCACCCAGCACAATGAA‐3′.

Human‐*Actin* (Reverse): 5′‐CTAAGTCATAGTCCGCCTAGAAGCA‐3′.

### 4.18 Statistical Analysis

Data are presented as mean ± standard deviation (SD). Unpaired two‐tailed Student's *t*‐test was employed for comparisons between two groups with equal variances, Welch's *t*‐test was employed for unequal variances, while the Mann–Whitney test was employed with non‐normal distribution. One‐way ANOVA followed by Tukey's multiple comparisons test was used for analysing differences among three or more groups, while the Kruskal‐Wallis test was used with non‐normal distribution. Two‐way ANOVA followed by Šídák's multiple comparisons test was performed for experiments with two independent variables. A value of *p* < 0.05 was considered statistically significant.

## Author Contributions

**Fa‐Ming Chen, Bei‐Min Tian**, and **Xuan Li** are the co‐corresponding authors. **Dao‐Kun Deng** contributed to the cell experiments, animal experiments, data analysis, data curation, figure preparation, writing – original draft, and writing – review and editing. **Meng‐Jie Su** contributed to the cell experiments, animal experiments, data analysis, data curation, figure preparation, and writing – original draft. **Min‐Yi Zhang** contributed to the cell experiments, animal experiments, data analysis, data curation, figure preparation, and writing – original draft. **Yu‐Jie Tan** contributed to the animal experiments, cell experiments, data analysis, data curation, and figure preparation. **Wenyao Kongling** contributed to the transcriptomic sequencing, bioinformatic analysis, in vitro infection experiments, data curation, and figure preparation. **Hong‐Yu Wang** contributed to the cell experiments and data curation. **Mei Xu** contributed to the cell experiments and data curation. **Shi‐Yu Liu** revised the manuscript and contributed to the writing – review and editing. **Xiao‐Tao He** contributed to data analysis and experiment design. **Fa‐Ming Chen** contributed to supervision, project administration, funding acquisition, and writing – review and editing. **Bei‐Min Tian** contributed to supervision, project administration, funding acquisition, and writing – review and editing. **Xuan Li** contributed to data analysis, data curation, figure preparation, supervision, project administration, funding acquisition, writing – original draft, and writing – review and editing.

## Funding

This work was supported by National Natural Science Foundation of China (82301079, 82571070, 82370957, 82571089, 82401186, 82401138), Natural Science Basic Research Program of Shaanxi Province (program no. 2024JC‐JCQN‐73), Chang Jiang Scholars Program—Young Scholar.

## Conflicts of Interest

The authors declare no conflicts of interest.

## Supporting information


**Data S1:** cpr70282‐sup‐0001‐Supinfo.pdf.

**Figure S1:** Concentrations of pro‐inflammatory cytokines (IL‐1β and TNF‐α) in the supernatants between control and *P. gingivalis* groups after another 24 h of incubation. Student's *t*‐test was employed with equal variances.
**Figure S2:** Expression of magnesium transporters and intracellular Mg^2+^ levels in control and *P. gingivalis* ‐infected macrophages. (A) The gene expression levels of *cyclin and CBS domain divalent metal cation transport mediator 1 (CNNM1)*, *CNNM2*, and *CNNM3* between the control and *P. gingivalis* groups; (B) The concentration of Mg^2+^ between the control and *P. gingivalis* groups.
**Figure S3:** The Mg^2+^ release curve of Alg‐Mg^2+^ within 35 days.
**Figure S4:** Representative Masson staining of periodontal tissue in control, periodontitis, Periodontitis + AlgMA, and Periodontitis + AlgMA‐Mg^2+^ groups. Black asterisks indicate the periodontal ligament (PDL). Alveolar bone (AB). Second molar (M2). Third molar (M3).

## Data Availability

The RNA sequencing data generated in this study have been deposited in the NCBI Sequence Read Archive (SRA) database under accession codes PRJNA1472389 (https://www.ncbi.nlm.nih.gov/bioproject/PRJNA1472389). All other data supporting the findings from this study are available within the Article and Supporting Information.
