# Supplementary material for: Magnesium Enhances Immune Clearance of P. gingivalis in Macrophages by Suppressing MCU‐Mediated Mitochondrial Calcium Overload
Source: Cell Prolif. 2026 Sep 25:e70282. Online ahead of print. doi: 10.1111/cpr.70282 (PMC13614854; doi:10.1111/cpr.70282)
Supplement: Supplementary file 2 [file CPR-9999-e70282-s001.docx]

**Magnesium Enhances Immune Clearance of *P. gingivalis* in Macrophages by Suppressing MCU-Mediated Mitochondrial Calcium Overload**

Dao-Kun Deng^1,2,#^, Meng-Jie Su^1,2,#^, Min-Yi Zhang^1,2,3#^, Yu-Jie Tan^1,2^, Wenyao Kongling^4^, Hong-Yu Wang^1,2^, Mei Xu^1,2^, Shi-Yu Liu^5^, Xiao-Tao He^1,2^, Fa-Ming Chen^1,2^, Bei-Min Tian^1,2^, and Xuan Li^1,2^

^1^State Key Laboratory of Oral & Maxillofacial Reconstruction and Regeneration, National Clinical Research Center for Oral Diseases, Shaanxi International Joint Research Center for Oral Diseases, Department of Periodontology, School of Stomatology, The Fourth Military Medical University, Xi’an, Shaanxi, 710032, China

^2^Military Medical Innovation Center, Fourth Military Medical University, Xi'an, Shaanxi, China

^3^Department of Periodontal and Oral Mucosa, Changsha Stomatological Hospital, Changsha, China

^4^Key Laboratory of Shaanxi Province for Craniofacial Precision Medicine Research, College of Stomatology, Xi’an Jiaotong University, Xi’an, 710049, China

^5^State Key Laboratory of Oral & Maxillofacial Reconstruction and Regeneration, National Clinical Research Center for Oral Diseases, Shaanxi Key Laboratory of Stomatology, Department of Oral Biology, School of Stomatology, The Fourth Military Medical University, Xi'an 710032, China.

Corresponding author: Xuan Li (li_xuan15@163.com). Xuan Li serves as the primary contact for proofs and publication. Fa-Ming Chen, Bei-Min Tian, and Xuan Li are co-corresponding authors of this paper.

Dao-Kun Deng ^#^, Meng-Jie Su ^#^, and Min-Yi Zhang ^#^ made equal contributions to this work.

**Supplementary Figure 1 Concentrations of pro-inflammatory cytokines (IL-1β and TNF-α) in the supernatants between control and *P. gingivalis* groups after another 24 h of incubation.** Student’s t-test was employed with equal variances.

**Supplementary Figure 2 Expression of magnesium transporters and intracellular Mg^2+^ levels in control and *P. gingivalis*-infected macrophages.** (**A**) The gene expression levels of *cyclin and CBS domain divalent metal cation transport mediator 1 (CNNM1)*, *CNNM2*, and *CNNM3* between the control and *P. gingivalis* groups; (**B**) The concentration of Mg^2+^ between the control and *P. gingivalis* groups.

**Supplementary Figure 3** The Mg^2+^ release curve of Alg-Mg^2+^ within 35 days.

**Supplementary Figure 4** Representative Masson staining of periodontal tissue in control, periodontitis, Periodontitis + AlgMA, and Periodontitis + AlgMA-Mg^2+^ groups. Black asterisks indicate the periodontal ligament (PDL). Alveolar bone (AB). Second molar (M2). Third molar (M3).
